# Oral Health-Related Quality of Life and Its Determinants

**DOI:** 10.3390/dj14100622

**Published:** 2026-09-29

**Authors:** Joanna Baginska, Magdalena Nowosielska

**Affiliations:** 1 Department of Dentistry Propaedeutics, Medical University of Bialystok, 15-295 Bialystok, Poland; 2 Department of Gerostomatology, Medical University of Bialystok, 15-295 Białystok, Poland; magdalena.nowosielska@umb.edu.pl

Traditional clinical indicators used in dentistry do not fully capture the impact of oral health on people’s daily functioning, emotional well-being and social interactions. Improving oral health requires patient-centered care that goes beyond the treatment of diseases and encompasses an improvement in overall well-being. This Special Issue, titled “Oral Health-Related Quality of Life and Its Determinants”, was created to bring together in one place articles on various aspects of research into quality of life related to oral health. This objective was achieved because each of the twelve articles, eleven research papers and one review, published in this Special Issue highlights a distinct perspective on quality of life assessment across different populations, age groups, and clinical situations, and they underscore the complexity of OHRQoL. One of the strengths of this Special Issue is its international perspective. The published studies come from various geographic regions, including Europe, Australia, the Middle East, and Asia, reflecting the global significance of research on OHRQoL.

The published papers address, among other topics, the oral health-related quality of life in individuals with chronic systemic diseases. Bruno et al. [1] assessed 90 patients with an acromegaly. Nearly half of them reported oral manifestations and most experienced facial changes, with poorer oral health-related quality of life being significantly associated with lower overall quality of life. Their study indicated that acromegaly has meaningful consequences for patients’ daily functioning and self-perception. Two studies involved diabetic patients. Alexa et al. [2] investigated determinants of oral health outcomes and OHRQoL among Romanian subjects with diabetes using Andersen’s Behavioral Model. Their findings demonstrate that socioeconomic status, health awareness, and access to dental care substantially influence both oral health status and patients’ perceived quality of life. A study showed that behavioral and social determinants were stronger predictors of OHRQoL than clinical parameters. João et al. [3] examined pediatric patients with type 1 diabetes mellitus and reported differences in oral manifestations, oral health behaviors, and OHRQoL compared with healthy peers. This study emphasizes that children living with chronic diseases require comprehensive dental care addressing both clinical needs and psychosocial well-being. Lansdown et al. [4] conducted a study among adults with cerebral palsy and found significant difficulties related to maintaining oral hygiene, accessing dental services, and receiving appropriate dental treatment. Functional limitations, communication difficulties, and socioeconomic factors contributed to a reduced quality of life related to oral health. Their study underscores the ongoing need to improve the accessibility and inclusiveness of dental care systems.

The influence of health behaviors and oral health literacy is represented by the study of Mishra et al. [5], who examined patients with cardiovascular disease. Their results demonstrate important gaps in oral health knowledge and preventive behaviors, suggesting that patient education should become an integral component of comprehensive cardiovascular care. This study also highlights the broader public health implications of integrating oral health promotion into medical care pathways.

Moroianu et al. [6] analyzed the course of psychological and social processes during dental treatment. They found that dental care was associated with improvements in patients’ psychological well-being and appearance-related satisfaction, indicating a positive impact on quality of life over the course of care. The authors concluded that psychosocial monitoring during dental treatment is feasible and may be beneficial. A German study conducted by Pampel et al. [7] also showed that the rehabilitation of severe oral function disorders has a positive impact on quality of life. In hospitalized geriatric patients, immediate prosthetic treatment improved chewing function and resulted in a high level of satisfaction with the treatment.

Children and adolescents constitute important populations represented in this collection. A study by Baty et al. [8] showed that the comprehensive dental treatment of children under general anesthesia, including cases requiring multiple extractions, leads to a significant improvement in oral health-related quality of life. Following treatment, improvements were observed not only in clinical status but also in functioning, accompanied by a reduction in the negative impact of dental problems on children and their families. According to the study of Alshaghdali et al. [9], the objectively assessed esthetics of the smile showed only a modest association with the psychosocial impact of dental appearance, whereas dissatisfaction with smile and tooth color was strongly associated with a lower quality of life related to oral health. These results suggest that, in routine dental care, subjective perceptions of dental esthetics should be taken into account alongside clinical assessments. Morgado et al. [10] demonstrated that children with asthma had poorer oral health compared to a control group of healthy individuals. Asthma remained an independent factor associated exclusively with low saliva secretion, while a history of toothache was the strongest predictor of poorer OHRQoL.

Simon et al. [11] explored the topic of relationships between dental esthetics, self-esteem, and oral hygiene habits, and the psychosocial issues in dental students. A more positive self-assessment of dental esthetics was associated with higher self-esteem, although differences were observed depending on gender and year of study. Regular checkups and the use of dental floss were also related to a more positive perception of dental appearance and self-image.

The most commonly used instrument in the articles published in the Special Issue was the Oral Health Impact Profile (OHIP). Wojszko et al. [12] provided a comprehensive summary of the available versions of the most commonly used tool for assessing oral health-related quality of life (OHRQoL) in adults, namely the Oral Health Impact Profile (OHIP). This study serves as a practical guide for researchers selecting appropriate tools. The authors emphasize the importance of careful cultural adaptation and encourage greater standardization in future studies on OHRQoL, facilitating comparisons across studies and populations. ECOHIS was predominant in pediatric studies, and specific instruments were used in special populations (e.g., PIDAQ for dental esthetics and AcroQoL for acromegaly).

In summary, the studies included in this Special Issue clearly demonstrate that quality of life related to oral health is a multidimensional concept influenced by the interplay of biological, psychological, behavioral, social, and environmental factors. Clinical indicators alone are insufficient to fully explain patients’ experiences. As Guest Editors, we hope that this Special Issue will contribute to a deeper understanding of oral health-related quality of life and stimulate further interdisciplinary research in this rapidly evolving field.

We would like to sincerely thank all the authors for their valuable contributions. Special thanks are given to the reviewers for their constructive feedback, which significantly enhanced the scientific value of the published articles. We extend special thanks to the Editorial Board of the *Dentistry* *Journal* for the support they provided us throughout the entire editorial process.
